# Supplementary material for: Are carbon emissions trading and green financial instruments synergistic? -Comprehensive quantitative research based on content analysis
Source: PLoS One. 2024 Mar 7;19(3):e0298601. doi: 10.1371/journal.pone.0298601 (PMC10919601; doi:10.1371/journal.pone.0298601)
Supplement: S1 Data — (ZIP) [file pone.0298601.s001.zip › Data/地级市Z_F工作报告.zip!/┴╔─■/╕╖╨┬╩╨/└┤╫╘╣1⁄2╓┌║┼í╛┬φ┐╦╩2╛▌═°í┐_2012.docx]

各位代表：

　　现在，我代表市政府向大会报告工作，请予审议，并请市政协委员和其他列席同志提出意见。

　　 一、2011年工作回顾

　　2011年，是阜新经济社会发展取得重大成就的一年。在市委的正确领导下，全市各族人民团结一心，奋力拼搏，胜利完成了市十四届人大四次会议确定的目标任务，实现了“十二五”高起点开局。

　　（一）经济实现快速发展，主要经济指标增幅位居全省前列

预计实现地区生产总值460亿元，增长16%，增幅全省第四；地方财政一般预算收入50.1亿元，增长66.8%，增幅全省第一；税收收入39.1亿元，增长52.1%，增幅全省第一；固定资产投资440亿元（新口径），增长71%，增幅全省第一；出口创汇1.45亿美元，增长20.8%，增幅全省第一；金融机构贷款余额435亿元，增长22.5%，增幅全省第一；城镇居民人均可支配收入15050元，增长18.4%，增幅全省第一；农村居民人均纯收入7500元，增长17.7%，增幅全省第二。

　　阜彰两县财政一般预算收入分别超过8亿元和6.5亿元，在全省位次前移；五城区财政一般预算收入实现翻番，均跨入3亿元行列。

　　 阜新经济实力增强，发展质量全面提升，在“十二五”开局之年进入了快速发展的新时期。

　　（二）项目建设取得突破，产业集群迅速发展壮大

“千个项目大会战”成效显著，“三大基地”和“六个重点产业集群”支撑作用增强。

煤化工产业呈现广阔前景，大唐煤制气一期全面开工，完成投资100亿元；大唐50万吨LNG和煤制气副产品深加工、香港华鼎70万吨LNG、中农集团煤化工、天赋军安煤制气深加工等一批重大项目前期工作启动。

新能源产业加快发展，新开工项目14个，风电装机容量达到180万千瓦，风电装备制造业销售收入实现56亿元。

　　农产品加工业进入新阶段，雨润肉类加工、辉山乳业等7个投资10亿元以上项目开工建设，项目数量居全省首位。振隆公司商标被认定为“中国驰名商标”。

　　液压产业发展态势强劲，新开工项目56个，销售收入突破200亿元，实现翻番，荣获“辽宁省示范产业集群”和“国家火炬计划特色产业基地”称号。

　　氟化工产业快速发展，实施项目31个，东欣氢氟酸等一批项目开工建设，浙江联化并购重组天予化工，香港理文氟化工等一批项目落户。

　　 皮革、板材家居、铸造、新型材料等产业加快推进，共新开工项目62个。

　　工业“五项工程”取得明显成效。规模以上企业增加65户，达到450户；销售收入超亿元企业增加23户，达到85户；阜矿集团总收入突破200亿元。民营经济增加值达到210亿元，增长41%，增幅全省第一。

　　科技创新步伐加快，高新技术产品增加值增长50%。

　　（三）城镇化进程加快，城市面貌发生显著变化

　　城市建设力度空前，资金投入、项目数量、建设速度、土地出让收益等实现历史性突破，城市框架拉大、品位提升。城镇化率提高2个百分点，达到52.5%。

　　新城建设和老城改造同步推进，全市房地产开发面积超过1000万平方米。

　　“玉龙新城”核心区路网框架基本形成，玉龙湖形成水面15万平方米，“两馆一心”、龙湖广场、大唐·荣成等12个项目开工建设，绿地集团等地产领军型企业进驻阜新。“沈彰新城”、“温泉旅游新城”等新城建设取得新进展。小城镇建设取得新成效。

　　 城市基础设施建设力度加大，完成中华路、解放大街、西山路、建设桥改造和中华路地下通道建设工程，城市外环路建设进程过半。阜盘高速建设进展顺利，彰通高速（阜新段）通车。

　　“五化工程”成果显著，市区植树673万株、栽摆鲜花695万株，新增绿化面积194万平方米；新建、改造街路30条，铺装人行步道超过50万平方米；拆除废弃建筑物191座，粉饰楼体119座；在城市出口路、市区重点街路、桥梁、沿街楼体等实施了新的亮化及其改造升级，消除了市区无灯路；城市环卫设施进一步完善，环境卫生状况明显改善，市容市貌变化较大。

　　建筑业产值超过150亿元，增幅全省第一。阜矿总医院急救中心综合楼获国家“鲁班奖”，填补辽西空白。

　　（四）招商引资成效明显，对外开放水平进一步提升

　　坚持“走出去”和“请进来”，全市招商引资到位300亿元，实现翻番，增幅全省第二；实际利用外资1.5亿美元，增长36.4%，增幅居全省前列。

　　重点围绕产业集群建设、新城开发、商贸物流发展，有针对性地洽谈推进项目，引进韩国斗山、鹰鹏集团、中国新兴建设集团等一批品牌企业；华润雪花、四川仙潭酒业等并购重组企业焕发新的生机；泰合风电、万利豪酒店等4个项目到位资金1亿美元以上；力达铸造、日本住林木业等企业出口创汇实现翻番。

　　成功举办首届中国液压装备制造业展览会、中国氟化工产业高峰论坛及篮球节、玛瑙博览会等活动，扩大了阜新知名度和影响力。

　　（五）农业增产增收，综合生产能力明显增强

　　粮食总产量超过50亿斤，创历史新高，阜彰两县被国务院授予“全国粮食生产先进县”称号。花生种植面积271万亩，跃居全国第三，阜蒙县成为全国花生第一县。

　　新建养殖小区476个，人均鲜奶占有量、人均肉羊和肉驴饲养量居全省第一。

　　农田水利投资12亿元，增长30%，新增及改善灌溉面积11.7万亩。滴灌节水农业工程开工面积38万亩，建设规模和质量全省领先。造林面积113万亩，200万亩荒山荒坡造林绿化工程全面启动。农田作业综合机械化水平达到73%，高于全省平均水平8个百分点。

　　（六）服务业发展步伐加快，重点行业实现升级

　　中央商务区成为我省首批现代服务业聚集区，时尚金街等4个项目投入使用，大润发、红星美凯龙等一批知名品牌落户阜新。商业开发档次提升，六和国际购物中心等6个城市综合体和鑫亚等4个五星级酒店开工建设。信息化发展步入新阶段，“无线城市”建设启动。社会消费品零售总额180亿元，增长20.8%，增幅居全省前列。

　　多渠道融资实现突破，域内金融机构新增贷款80亿元，域外银行融资规模突破100亿元；阜新银行大连分行开业；组建小额贷款公司96家，数量居全省前列。新增存款98亿元，增长20%。

　　瑞应寺晋升为国家4A级旅游景区，海棠山红石谷景区开园，温泉旅游开发成为热点。旅游总收入70亿元，增长30%。

　　（七）发展成果惠及百姓，民生质量持续提高

　　向全市人民承诺的15件实事全部兑现。

　　 棚户区改造走在全省前列，建成回迁楼315万平方米，10万多群众居住条件得到根本改善。“城中村”、“城边村”改造使1.1万户居民受益。

　　实名制就业6.3万人次。省级创业型城市创建工作进展顺利，首届创业文化节激发了市民创业热情。最低工资标准由每月650元提高到780元。

　　 覆盖城乡的社会保障体系进一步完善。新型农村和城镇居民社会养老保险全面推开，参保率均超过80%；企业退休人员基本养老金月人均提高130元；新型农村合作医疗参合率93%，比上年提高10个百分点。

　　老旧住宅小区环境综合整治工程三年任务超额完成，当年整治面积182万平方米。对80万平方米居民楼进行供热计量和节能改造。新建市民休闲广场50个、健身步道50条。城市环境空气质量二级以上标准天数达标率95%以上。

　　农村10所初中进县城，新建、改扩建幼儿园26所，维修改造校舍38万平方米，建设农村学校塑胶操场16块。

　　18.6万农村贫困人口年人均纯收入由1500元提高到1800元以上。解决12万农村人口饮水安全问题，新建农村无害化厕所1.5万座。

　　启动社会救助和保障标准与物价上涨挂钩联动机制。低保户取暖救助标准提高100元以上。“百户慈善建房”和减灾安居工程让163户困难居民喜迁新居，我市荣获“首届中国城市公益慈善指数”最高奖。

　　（八）社会建设全面推进，社会管理得到加强

　　海州区、细河区通过“双高普九”验收，中小学教育信息化“班班通”工程启动，二职专新校区建设工程主体完工，市校共建的辽宁工大国家大学科技园启动。

　　市民文明素质和社会文明程度“双提升”工程有效实施，各类文化活动蓬勃开展，文化体制改革取得新进展。

　　基层医疗卫生机构综合改革走在全省前列，城乡医疗水平整体提升。省级卫生城通过复检，全国首家市级人口与家庭公共服务中心成立。

　　群众体育与竞技体育同步进展，我市被国家体育总局确定为东北三省唯一的“全民健身示范城市”试点市。

　　社会矛盾大排查大调处活动深入开展，一批信访问题得到妥善化解。社会治安防控体系进一步完善，打击刑事犯罪工作卓有成效，群众安全感普遍提升。安全生产工作扎实推进，各类隐患得到有效整治，总体形势保持平稳。

　　军分区办公楼等双拥项目加快推进。我市连续七年荣获省双拥模范城称号。民族团结进步事业深入发展。第八届社区居委会换届选举完成。第六次全国人口普查工作获国家奖励。

　　 政府自身建设进一步加强。“行政效能大提升”活动成效明显，促进了政府职能和作风转变。精简下放管理权限和行政审批事项30项。坚持向市人大及其常委会报告工作和向市政协通报重要情况制度，办复人大代表建议138件、政协提案152件。行政权力运行制度系统建设稳步推进，市本级行政权力电子监察系统建成使用，反腐倡廉建设取得新成效。

　　过去的一年，全市各行各业、各条战线都呈现了新变化，取得了新业绩。特别是中省直驻阜单位坚持以振兴阜新为己任，充分履行职能，积极献计出力，为地方经济社会发展作出了重要贡献。

　　各位代表，2011年阜新的发展变化有目共睹、振奋人心。这些成绩的取得，是省委、省政府重视关怀、大力支持的结果，是市委科学决策、正确领导的结果，是市人大、市政协监督支持的结果，是全市人民共同奋斗的结果。在此，我代表市政府，向全市各族人民，向人大代表、政协委员，向各民主党派、工商联、无党派人士和各人民团体，向中省直单位、驻阜部队和武警官兵，向离退休老领导、老同志，向所有为阜新发展作出贡献的同志们、朋友们，致以崇高的敬意和衷心的感谢！

　　在看到成绩的同时，我们也清醒地认识到，全市经济社会发展中还存在一些矛盾和问题。主要表现在：经济总量不足的状况还没有根本改变，做大总量、做强财力的任务仍很艰巨；产业层次不高，现代产业体系建设步伐不快；实际利用外资规模较小，缺乏牵动作用大的龙头项目；土地、资金等要素制约趋紧，节能减排压力不断增大；城镇化水平较低，城市基础设施承载能力不足；部分群众生活还比较困难，保障和改善民生的任务仍很繁重；一些机关干部服务意识不强，办事效率不高，能力素质与新形势新任务要求不相适应。对于这些矛盾和问题，我们要高度重视，采取有力措施，认真加以解决。

　　二、2012年工作安排

　　2012年，是我市创建转型示范市、实现加快发展的关键之年。按照市委十一届二次全会部署，政府工作的总体要求是，全面贯彻落实科学发展观，以创建转型示范市为主线，以做大总量、提高城乡居民收入水平为目标，深入实施“工业强市、城镇化带动、开放创新、生态立市、文化兴市”战略，加快转变经济发展方式，大力推进工业集群化、新型城镇化、农业产业化进程，全面提升综合经济实力，实现经济快速发展、社会事业全面进步、人民生活更加幸福。

　　主要预期目标是：地区生产总值580亿元，增长18%；地方财政一般预算收入64亿元，增长28%；固定资产投资570亿元，增长30%；社会消费品零售总额210亿元，增长17%；城镇居民人均可支配收入17800元，增长18%；农村居民人均纯收入8700元，增长16%。

　　在实际工作中，为了体现加快发展，我们不仅要必保完成上述目标，而且要超额完成，力争主要经济指标增幅继续保持全省前列。我们就是要通过超常努力，让全市经济发展得更快、城市建设得更美、人民生活得更好。

　　为此，我们要围绕财政抓经济、围绕民生抓发展，全年工作的主基调是“稳中求快、又快又好”，主要抓手是经济增长点，主要措施是向上争、对外招、奋力干。我们就是要用各级干部的辛苦指数换取人民群众的幸福指数。

　　（一）强力推进项目建设，为加快发展提供强大基础

　　把项目建设作为经济发展的重中之重，以“项目建设年”活动为载体，围绕壮大“三大基地”和“六个重点产业集群”，集中精力抓项目建设，实现投资大增加，拉动经济大发展。全年新开工投资千万元以上项目400个，续建600个。

　　加快煤化工基地建设，大唐煤制气一期第一系列投产，开工建设中农集团煤化工、大唐LNG、香港华鼎LNG、北京国能煤焦油等项目，推进大唐煤制气二期、天赋军安煤制气深加工等项目前期工作。巴新铁路试通车。

　　加快新能源产业发展，风电装机容量超过200万千瓦，开工建设中石化雷家油田、中国风电光伏电站等重点项目，杰超热电实施搬迁改造。

　　加快农产品加工项目建设，雨润肉类加工、辉山乳业等重点项目建成投产，推进娃哈哈、维维集团等知名企业落户阜新。

　　推动液压产业集群向高端化、品牌化方向发展，开工建设通用电梯、韩国斗山液压机床等80个项目，尤尼深风机设备制造、金风风电设备制造二期等一批项目投产。

　　进一步提高皮革产业集群深加工能力，新开工项目30个，重点推进富国皮革、辽宁富新等汽车革项目，宏均皮革、东亚皮革等沙发革项目，雅尊服饰制衣、斯沃德裘皮等服装制品项目。

　　加大氟化工产业集群新产品开发力度，重点推进香港理文氟化工、浙江联化医药中间体等20个项目开工建设，东欣氢氟酸、恒通氟碳醇等一批项目投产。

　　加快铸造产业集群向百亿元目标迈进，开工建设太克集团铸造深加工、力劲铸造二期、金达铸件等16个项目，冰轮机床铸造等一批项目投产。

　　提升板材家居产业集群水平，开工建设舒丽雅家具、日本住林木业二期等22个项目，赛斯木业等一批项目投产。

　　做大新型材料产业集群，开工建设香港天溢罗布麻纺织、上海矿岩棉保温材料等32个项目，保利民爆等项目投产。

　　健全完善项目工作责任制，对全年实施的项目层层落实任务、落实措施、落实责任。强化项目推进保障机制，坚持月调度、季通报、年终考核制度，对重点项目全程跟踪督查，尽最大努力保障土地、资金等要素供给。提高项目建设质量和标准，严格考核项目开工率、竣工率和投产率，努力使项目建设周期最短、速度最快、效率最高。

　　（二）大力实施“工业强市”战略，提升工业经济整体实力

　　全力抓好产业集群、企业提升、项目建设、并购重组和节能降耗等“五项工程”，促进我市工业发展再上新台阶。

　　深入实施企业“倍增计划”，全年倍增企业达到100户；销售收入超亿元企业突破100户；规模以上企业达到530户，实现增加值210亿元，增长20%以上。阜矿集团总收入超过240亿元。

　　鼓励和支持企业技术改造，推动企业上市融资。完善中小企业融资担保体系，扶持一批成长性好、潜力大的中小企业快速发展。

　　加快园区基础设施建设，完善配套服务平台，提高承载能力。进一步优化产业布局，拓宽园区发展空间。

　　大力推进科技创新，加快各产业集群研发中心建设，健全科技服务体系。深化与高等院校、科研院所合作，扩大与辽宁工大在科技研发、人才培养、社会管理创新等方面的合作成果。大力培育科技创新型、领军型企业，提高企业核心竞争力。全市高新技术产品增加值增长50%以上。

　　（三）坚持新城建设与老城改造并举，加快推进城镇化进程

　　围绕城市总体规划，坚持高标准建设、高水平开发、高速度改造，提高城市综合承载能力和建设品位。

　　加快新城开发建设。“玉龙新城”开发面积达到300万平方米，核心区道路、桥梁等基础设施基本完成，行政服务中心、企业总部等一批项目开工，重点打造绿地·剑桥、大唐·荣城等标志性工程。进一步完善新城功能，启动商业中心区和商务核心区建设。“沈彰新城”、“温泉旅游新城”、“现代皮革新城”、“蒙古贞新城”建设要有新形象。

　　加快城市中心区危旧楼改造，推进站前、孙家湾等南部地区开发，促进老城改造升级。

　　提升县城建设水平，阜彰两县房地产开工面积突破100万平方米。抓好省市两级城镇化试点，积极、稳妥、有序地推动农民进城。

　　在新城建设和老城改造中，全面推进住宅、商业、工业、旅游、教育、文化等地产开发，全年地产开工面积1000万平方米以上。建筑业产值超过200亿元，增长30%以上。

　　新建和改造城市道路100公里以上，城市外环路、沈彰开发大道、新邱开发大道、皮革产业大道竣工通车。阜盘高速建成通车，推进沈阜直通高速项目。

　　全面加强城市环境卫生管理，实现主要街路清扫机械化、保洁全天化、监管常态化。加强绿地及公用设施管理，集中开展违章建筑、占道经营、露天餐饮、户外广告等专项整治。实施小区物业标准化建设，不断提升物业管理水平。

　　（四）狠抓招商引资，进一步扩大对外开放

　　越是在宏观调控趋紧的形势下，越要招商引资、扩大开放，更多地引进资金、项目、技术、管理和先进理念。要大规模“走出去”、“请进来”，着力引进带动能力大、税收贡献多、成长性好、科技含量高的项目，提高对外开放水平。

　　继续突出主题概念、产业集群招商，面向珠三角、长三角、日韩、香港等重点国家和地区，大力推进点对点、面对面等深度招商。坚持专业队伍招商，创新以商招商和委托招商等方式，提高招商的针对性和实效性。继续开展项目集中签约活动，提高阜新的影响力和吸引力。全年招商引资到位400亿元以上，增长30%以上。

　　提高利用外资水平，扩大工业产业集群、服务业、新兴产业等领域引进外资的规模。实际利用外资超过2亿美元，增长30%以上。

　　调整优化出口产品结构，加快培育骨干企业，出口创汇1.9亿美元，增长30%以上。

　　深入实施沈阳经济区一体化战略，加快推进沈阜经济带建设，提升区域合作水平。

　　（五）大力发展县域经济，促进两县富民升位

　　加快县域“三化”进程，扎实推进社会主义新农村建设。继续实施县域经济“三年倍增”计划，阜彰两县财政一般预算收入、固定资产投资都要增长40%以上。

　　以产业集群化为重点，加快县域工业和服务业聚集区建设，两县力争形成一个产值超50亿元的产业集群和一个现代服务业聚集区。

　　以现代农业为重点，加快农业结构调整。巩固花生种植面积，做大“一县一业”品牌。加快发展设施农业。进一步提高畜牧业规模化饲养水平，新建养殖小区300个。加快农业产业化进程，扶持各类龙头企业发展，提高农产品精深加工水平，同时做大做强农产品生产基地。推进土地承包经营权流转，促进土地规模化经营。加强农业科技支撑体系建设，培育一批现代农业示范区。进一步完善动物疫病防控体系、农产品质量安全监管体系，提升农产品质量安全水平。

　　以农田水利建设和监管为重点，提高农业综合生产能力。高标准完成滴灌节水农业一期工程；实施中低产田改造、云水资源开发等工程；做好辽西北供水工程配套服务工作；提高农田作业综合机械化水平。加强农村道路建设、电网改造，推进农村环境综合整治。

　　以经济发展、财政增收、农民致富为目标，制定和实施乡村经济发展规划，重点打造一批财政收入超3000万元的乡镇和经营收益超100万元的村。

　　（六）大力发展城区经济，提升现代服务业水平

　　深化城区“两项改革”，启动新一轮城区经济“三年倍增”计划，促进服务业与工业聚集区齐头并进。五城区财政一般预算收入、固定资产投资都要增长40%以上。在城区街道建立财政收入奖励机制，充分调动街道抓项目、抓收入的积极性。

　　加快打造服务业聚集区，开工建设兴隆CBD和大润发、红星美凯龙、沃尔玛等大型商场，中央商务区实现销售收入100亿元。站前、孙家湾、开发区“金廊”等服务业聚集区初具规模。

　　积极发展物流、信息等新兴产业。推进北方国际物流园、锦州港国际集装箱物流园、交通物流中心等项目建设。发展农村现代流通方式，降低农产品流通成本。加强专业市场建设，启动百联汽配城、五金建材城等一批项目。整合全市信息资源，加快 “无线城市”建设步伐。

　　创新融资方式，做大间接融资、拓宽直接融资、壮大市场融资、激活民间融资，促进金融业快速发展。全年融资额度达到260亿元，交通银行阜新分行、汇通投资基金公司正式运营。

　　进一步完善瑞应寺、国家矿山公园、大清沟等重点旅游景区基础设施，启动实施海棠山旅游经济开发区、关山敖包旅游风景区、玛瑙文化创意产业园、温泉小镇等一批项目，加快旅游业发展。

　　（七）加强生态环境建设，努力打造宜居城市

　　扎实推进生态文明建设，促进资源合理开发、环境有效保护、生态持续改善、城乡更加宜居。

　　实施青山工程，加快矿山治理和生态恢复步伐，重点抓好高德矸石山生态治理工程。加快荒山荒坡造林绿化，力争三年任务两年完成。突出发展特色优质水果、文冠果等经济林，全年造林120万亩。

　　实施碧水工程，落实大小凌河及辽河干支流治理计划，抓好细河综合治理，养息牧河治理达到省验收标准。统筹水资源开发、管理、保护，启动水务一体化改革。

　　实施蓝天工程，推进集中供热，推广清洁能源，继续开展大气污染整治工作，拆除禁燃区内各类燃煤设施。坚决完成节能减排任务。

　　加大城市绿化力度，打造一街一品、一路一景的绿化景观，城区栽植树木700万株、鲜花750万株，新增绿化面积200万平方米。积极创建国家园林城和卫生城。

　　大力发展循环经济，加快再生资源循环经济产业园建设，新建10条煤矸石、粉煤灰综合利用生产线。积极推进海州露天矿抽水蓄能电站项目前期工作。

　　（八）认真抓好百项民生工程，努力提高人民群众的幸福指数

　　强化民生优先的理念，把民生这条主线贯穿于政府的一切工作之中，以深厚的感情关注民生，以高度的责任保障民生，以最大的努力改善民生，让人民群众的日子过得越来越好。

　　全面实施百项民生工程，加大投入力度，大幅度增加惠民成果。今年，市政府要办好一批直接关系民生、反映民愿的实事。

　　1．实现实名制就业5万人次，开展创业培训2000人。

　　2．新建城市棚改回迁住房2000套；解决低收入家庭廉租住房等2600户；实施农村慈善建房200户。

　　3．改造4个“城中村”和2个“城边村”。

　　4．实施居民楼节能改造1.4万户、100万平方米。

　　5．新增煤气用户8000户，解决4000户城市居民吃水难问题。

　　6．新增城市水系面积30万平方米。

　　7．新建城市公园绿地5处，改造市区7个广场绿化景观。

　　8．建成市垃圾处理场二期和10个乡镇污水处理厂。

　　9．新建农村饮水安全工程100处，解决6万人饮水安全问题。

　　10．市区新建和改造公厕20座，农村改厕1万座。

　　11．新建、改造城市街路43条，铺设人行步道30万平方米，对20条街路进行亮化改造升级。

　　12．改造村屯道路200公里。

　　13．“两馆一心”主体完工。

　　14．解决80个自然屯广播电视覆盖盲点问题。

　　15．建设1万平方米的多功能市老年活动中心。

　　16．向市民免费开放市人民公园、图书馆、博物馆。

　　17．参加医疗保险居民门诊统筹报销比例提高10%，门槛费降低20%；实现城镇基本医疗保险市级统筹及“一卡通”结算。

　　18．新建农村学校塑胶操场16块。

　　19．新建、改扩建幼儿园19所。

　　20．新建农村社区服务中心60个。

　　21．新建城市社区“农超对接”蔬菜直销点50个。

　　22．实名安置500名残疾人就业。

　　23．推动100户企业实行工资集体协商。

　　24．企业退休人员基本养老金提高10%；提高城乡居民最低生活保障标准和农村五保对象供养标准。

　　25．新增农村沼气用户2000户。

　　26．建设和维修10座垂直压缩式垃圾转运站。

　　27．建设村文化广场268个，实现全覆盖。

　　28．市中心医院新建的老干部病房投入使用。

　　29．为50%以上的城乡居民建立规范化电子健康档案。

　　30．加大扶贫开发力度，使13万农村低收入群体年人均纯收入达到2200元以上。

　　在这里特别强调的是，今年新增财力将重点用于改善民生。为了老百姓的福祉，我们要以真挚的感情、公仆的本色，殚精竭虑，尽其所能，将实事办好、好事办实，我们一定会，也一定能兑现全部诺言。

　　（九）加快发展社会事业，提升社会管理科学化水平

　　 深入实施“文化兴市”战略，注重用文化凝聚力量、引领方向。继续实施“双提升”工程，广泛开展群众性文化活动，办好玛瑙博览会等品牌项目，推出更多的文化精品。完善城乡公共文化服务体系，开工建设　　市传媒大厦等项目。深化文化体制改革，促进文化事业繁荣和文化产业发展。

　　抓好人才培养、引进和使用工作，保障重点产业人才需求，启动“引博入阜”等高层次人才引进工程，着力营造尊重劳动，尊重知识，尊重人才，尊重创造的良好氛围。

　　推进阜彰两县通过“双高普九”验收，重视发展学前教育，开展义务教育均衡发展示范县区创建工作。市实验中学启动跨地区招生，开工建设二部综合教学楼。加快发展职业教育。实施校园安全工程，强化校车管理。加强教师队伍建设，提高整体素质。

　　深化基层医疗卫生机构综合改革及基本药物制度改革，进一步提高城乡医疗水平。加强医疗卫生基础设施建设，市中心医院、精神病医院、第五人民医院等住院大楼交付使用，开工建设阜矿总医院内科大楼。深化人口和计划生育综合改革，全面推进“幸福家庭促进计划”，促进人口均衡发展。

举办市首届全民健身运动大会，推进“篮球城”建设向更高层次迈进，扎实创建国家“全民健身示范城市”。积极做好“十二运”有关筹备工作。

　　加强和创新社会管理，完善维护群众权益机制、群众诉求表达机制和社会矛盾调处机制，健全社会矛盾排查化解、基层社会管理服务体系，更好地协调社会关系。加强和改进社区工作，不断完善社区服务体系。支持和保障司法机关依法独立行使职权，维护社会公平正义。用群众工作统揽信访工作，着力解决群众反映强烈、影响社会和谐稳定的突出问题。

　　认真落实市人大及其常委会的决议和决定，自觉接受人大法律监督和政协民主监督，主动听取各民主党派、工商联、无党派人士及各人民团体的意见，广泛接受社会公众和新闻舆论监督。

　　深化平安创建活动，加强社会治安综合治理，严密防范和依法打击各类违法犯罪活动。落实安全生产主体责任，保持安全生产形势平稳。加强食品药品监管，保障人民群众饮食和用药安全。创新双拥方式，深化双拥共建工作。深入开展“六五”普法工作，提高全民法律素质。认真落实党的民族宗教政策，促进各民族共同团结奋斗、共同繁荣发展。

　　（十）着力加强自身建设，打造人民满意的政府

　　新形势新任务对政府工作提出了新的更高要求。我们要以开展“改革创新年”活动为契机，大力推进政府职能转变和管理创新，切实提高政府工作水平，肩负起加快发展的历史重任。

　　我们要勇于改革创新。把改革创新作为加快发展的不竭动力，不唯思维定势，不唯发展模式，只唯发展实际。将加强学习作为改革创新的重要前提，让广大干部创造活力竞相迸发。勇于深化各领域改革，敢于放开一切可以放开的领域，坚决消除一切阻碍发展的体制机制，激发工作动力，释放发展活力。

　　我们要满怀发展激情。一个地区、一个单位的发展水平和成效，往往取决于干部的精神状态。在阜新发展的关键时期，我们要善于乘势而为，敢于逆势而上；无论遇到什么困难，我们都要坚定信心，不气馁、不动摇、不懈怠，加快发展、加快追赶；要以科学的态度、务实的作风，以只争朝夕的干劲、奋发有为的精神，干事创业，攻坚克难，不甘落后，敢为人先。

　　我们要提高工作效能。深入开展“行政效能大提升”活动，着力整治行政不作为、滥作为、慢作为现象。要做到事不避难，事不过夜，立说立行，行必有果。保持工作高强度、快节奏，往前抢、争主动，努力营造办事最快、成本最低、服务最优的政务环境。

　　我们要更加廉洁从政。廉洁是政府机关的第一形象。要深入推进“五大系统”建设，更加注重从源头上惩治和预防腐败。认真解决损害群众利益的突出问题，坚决查处违法违纪案件。加强公务员队伍建设，提高公务员整体素质，努力塑造为民、务实、清廉的形象。

　　各位代表，新的目标催人奋进，新的跨越时不我待。让我们在市委的领导下，凝聚全市人民的智慧和力量，坚定信心，再接再厉，为全面完成2012年各项任务、加快创建转型示范市而努力奋斗！我们坚信，阜新的明天一定会更加美好，阜新人民的生活一定会更加幸福！
